# Guidance impact on primary care prescribing rates of simple analgesia: an interrupted time series analysis in England

**DOI:** 10.3399/bjgp20X714101

**Published:** 2021-02-23

**Authors:** Hannah Reichel, Rhian Stanbrook, Hans Johnson, William Proto, Mary Shantikumar, Pooja Bakhshi, Sarah Hillman, Dan Todkill, Saran Shantikumar

**Affiliations:** Warwick Medical School, University of Warwick, Coventry.; Medwyn Surgery, Surrey.; Bristol Medical School, University of Bristol, Bristol.; Warwick Medical School, University of Warwick, Coventry.; Central Surgery, Rugby.; Registrar in paediatrics, Health Education West Midlands, Birmingham.; Warwick Medical School, University of Warwick, Coventry.; Warwick Medical School, University of Warwick, Coventry.; Warwick Medical School, University of Warwick, Coventry.

**Keywords:** analgesia, general practice, interrupted time series analysis, prescriptions

## Abstract

**Background:**

In March 2018, NHS England published guidance for clinical commissioning groups (CCGs) to encourage implementation of policy to reduce primary care prescriptions of over-the-counter medications, including simple analgesia.

**Aim:**

To investigate the impact of guidance publication on prescribing rates of simple analgesia (oral paracetamol, oral ibuprofen, and topical non-steroidal anti-inflammatory drugs) in primary care; CCG guidance implementation intentions; and whether the guidance has created health inequality based on socioeconomic status.

**Design and setting:**

Interrupted time series analysis of primary care prescribing data in England.

**Method:**

Practice-level prescribing data from January 2015 to March 2019 were obtained from NHS Digital. Interrupted time series analyses were used to assess the association of guidance publication with prescribing rates. The association between practice-level prescribing rates and Index of Multiple Deprivation scores before and after publication was quantified using multivariable Poisson regression. Freedom of information requests were submitted to all CCGs.

**Results:**

There was a statistically significant 4.4% reduction in prescribing of simple analgesia following guidance publication (adjusted incidence rate ratio 0.96, 95% CI = 0.92 to 0.99, *P* = 0.027), adjusting for underlying time trend and seasonality. There was considerable diversity across CCGs in whether or how they chose to implement the guidance. Practice-level prescribing rates were greater in more deprived areas.

**Conclusion:**

Guidance publication was associated with a small reduction in the prescribing rates of simple analgesia across England, without evidence of creating additional health inequality. Careful implementation by CCGs would be required to optimise cost saving to the NHS.

## INTRODUCTION

In light of the current funding deficit in the NHS, it is imperative that spending is made more efficient^1^ — a sentiment acknowledged by *The NHS Long Term Plan* published in 2019.^2^ One previously identified key area for improvement is medication optimisation: ensuring medicines are both clinically effective and cost-effective.^3^ Pharmaceutical spending is a common source of financial strain on healthcare systems worldwide, and is one of the highest NHS expenditures, second only to staffing.^4^ NHS England published guidance in March 2018 specifying medications that should not be routinely prescribed in primary care, including items that are available for purchase over the counter.^5^ While this guidance allows for a nationally coordinated response, the decision to implement it as a policy, as well as the choice of implementation strategies, lies with clinical commissioning groups (CCGs) — statutory regional NHS bodies that are responsible for the planning and commissioning of healthcare services for their local area.^6^

Before publication of the prescribing guidance, stakeholder consultation revealed a fear that implementation could perpetuate health inequalities given the consequent need for people to purchase some medications themselves over the counter (OTC), which some individuals may not be able to do. Subgroups thought to be at particular risk were people with disability, older people, those of lower socioeconomic status, or those with a limited capacity for selfcare.^7^

The estimated annual spend across the NHS on simple analgesia for minor conditions associated with pain, discomfort, or fever is 38 million GBP,^5^ or around 7% of total spending on OTC medication in the year before 2017. The recent NHS England guidance suggests that people should be encouraged to supply their own OTC analgesics for minor conditions such as colds, earache, teething pain, and self-limiting musculoskeletal pain; including those who would normally be exempt from paying the usual prescription charge in England, such as those aged <16 years or >60 years, pregnant females, individuals on income support, and those with one of a specified list of medical conditions. Patients in England pay a fixed per-item prescription charge, which does not necessarily cover the total cost incurred by the NHS in prescribing these medications. However, for those exempt from paying prescription charges, a requirement to purchase their own OTC medications will result in a personal cost.

The aim of this study was to evaluate the impact of the March 2018 NHS England guidance^5^ on primary care prescribing of simple analgesia available OTC: paracetamol tablets and suspensions; ibuprofen tablets and suspensions; and topical non-steroidal anti-inflammatory drugs (NSAIDs), as identified in the guidance. Specifically, the authors aimed to: explore whether there has been a change in the prescribing rates of simple analgesia since the publication of the guidance; explore the extent to which individual CCGs have considered and implemented this guidance; and assess whether there is any evidence the guidance has resulted in a health inequality by socioeconomic deprivation.

**Table:** How this fits in

As part of a medication optimisation strategy, in March 2018 NHS England published guidance for clinical commissioning groups (CCGs) that included a list of over-the-counter medications that should not be routinely prescribed by GPs in the NHS. Specifically examining simple analgesia, such as paracetamol and ibuprofen, the authors found only a small reduction in national prescribing rates following guidance publication. Information collected through freedom of information requests to CCGs found a diverse approach to guidance implementation, with some areas having no plans for implementation. The findings suggest that guidance publication alone had little benefit in reducing prescribing rates. Careful implementation would be required to achieve the full potential cost-saving benefit of the guidance to the NHS, though care needs to be taken to ensure that implementation does not result in health inequality, with the patients having to purchase medication items themselves.

## METHOD

### Description of the guidance

NHS England published the document, *Conditions for Which Over the Counter Items Should not Routinely be Prescribed In Primary Care: Guidance for CCGs*, in March 2018.^5^ Aimed at CCGs, this guidance includes items that can be purchased OTC, often at a lower personal cost than that which would be incurred by the NHS (in part due to additional administrative and dispensing costs), as well as medications that lack robust evidence for clinical effectiveness. Some drug classes are subject to specific exceptions where they may justifiably be prescribed, for example, it is suggested that vitamins are not prescribed except where there is a medically diagnosed deficiency, osteoporosis, or malnutrition. The guidance also provides a list of ‘general exceptions’ — criteria where the guidance need not apply and OTC medication may be prescribed by the primary care physician. These exceptions include where patients are prescribed a medication for long-term conditions (such as chronic arthritis), where patients have complex medical issues (such as immunosuppression), or where a medication is being prescribed for an unlicensed indication.

### Data sources

Primary care prescribing data in England are published by NHS Digital (https://digital.nhs.uk) on a monthly basis, detailing the number of items, quantity, and cost of NHS prescriptions dispensed in the community by individual primary care practices.^8^ Monthly datasets were downloaded from January 2015 to March 2019, up to 12 months after the publication of the NHS England guidance, hereby also referred to as the ‘intervention’.

A list of *British National Formulary* (BNF) codes was curated for each of the simple analgesics mentioned in the NHS England policy (Supplementary Box S1).^9^ Specifically, this included paracetamol tablets (up to 500 mg), paracetamol suspensions, ibuprofen tablets (up to 400 mg), ibuprofen suspensions, and topical NSAIDs, and excluded opioid medications. Branded and generic medications were included. Prescription-only medications, and those combined with other drugs (such as co-codamol), were excluded. The monthly prescribing datasets were filtered, by BNF code, to include only simple analgesia.

The number of items of simple analgesia prescribed by each practice every month was aggregated. Information on age, sex-stratified practice list sizes, published quarterly by NHS Digital,^10^ was retrieved to calculate the monthly prescribing rate per 1000 patients. Practice-level socioeconomic deprivation data, as quantified by the Index of Multiple Deprivation (IMD) score,^11^ were retrieved from Public Health England,^12^ recoded as quintiles, and linked to prescribing data as previously described.^13^

### Interrupted time series analysis

Interrupted time series analyses (ITSAs) were conducted using segmented Poisson regression to elicit an effect of the intervention on primary care prescribing, with the number of items prescribed per month as the dependent variable, using the total GP-registered population as an offset variable to model rates.^14^ The ITSA model includes month as a linear variable to model for an underlying linear time trend (with month in the dataset labelled from 1 to 51, for the 51 monthly prescribing datasets used), and the intervention as a dummy variable, coded ‘0’ for the pre-intervention period and ‘1’ for the post-intervention period. A second (adjusted) model additionally accounted for seasonality in the underlying prescribing rates, using a harmonic term based on the month of the year and using two sine/cosine pairs per 12-month period.^14^^,^^15^ Initial analyses suggested overdispersion of data, so a quasi-Poisson model was used. It was hypothesised that the intervention would result in a level (step) change in the outcome, given how widely the NHS England guidance was reported at the time of publication.^16^^,^^17^ Any changes in linear trend after this point would likely be affected by how well the guidance was subsequently implemented, so the authors did not include an analysis of this in the present model. The pre-intervention time period was from January 2015 to March 2018, and the post-intervention time period was from April 2018 to March 2019 for this analysis,. There were no documented missing data in the NHS Digital prescribing or practice list size data, and no sensitivity analyses were conducted.

### Association with deprivation

The association between practice-level IMD score and annual simple analgesia prescribing rates, 12 months before and after the intervention, was tested using univariate and multivariable Poisson regression, the latter adjusted for the practice proportion of males, proportion of those aged >65 years, and practice list size, as the authors have previously found practice age and sex distribution, and practice list size to be confounders for practice-level prescribing of other medications.^13^ Poisson regression analyses are presented as unadjusted (IRRs) or adjusted incidence rate ratios (aIRRs), comparing the relative rate of prescribing in each IMD score quintile with practices in quintile 1 as the reference group (the least deprived quintile). CCG-level prescribing was stratified by deciles and plotted on a choropleth map, with the use of CCG boundary shapefiles published by the Office for National Statistics^18^ to visualise geographic disparity in prescribing rates. The pre-intervention time period was from April 2017 to March 2018 and the post-intervention time period was from April 2018 to March 2019 for this analysis.

A *P*-value <0.05 was considered statistically significant. All data were analysed, and all plots generated, using R (version 3.5.3). The template R script is available at https://github.com/sirsazofduck/2020ReichelH.

### Freedom of information requests

A freedom of information (FOI) request was submitted to all 191 CCGs (as of April 2019) for information concerning their level of consideration and implementation of the NHS England policy, and the prescribing of analgesics available OTC (see Supplementary Box S2 for the full list of questions). As there was considerable diversity in the methods and strength of implementation (from ‘position statements’ to local guideline development, with or without additional education or incentives) as well as in the timing of implementation, which in some cases occurred before the publication of the national guidance, the authors were not able to examine whether or not the strength of implementation was associated with the level magnitude or trend of change of prescribing rates. A qualitative analysis of the CCG responses is outside the scope of the current study and will be conducted separately.

## RESULTS

### Trends in prescribing rates

**Figure 1.**
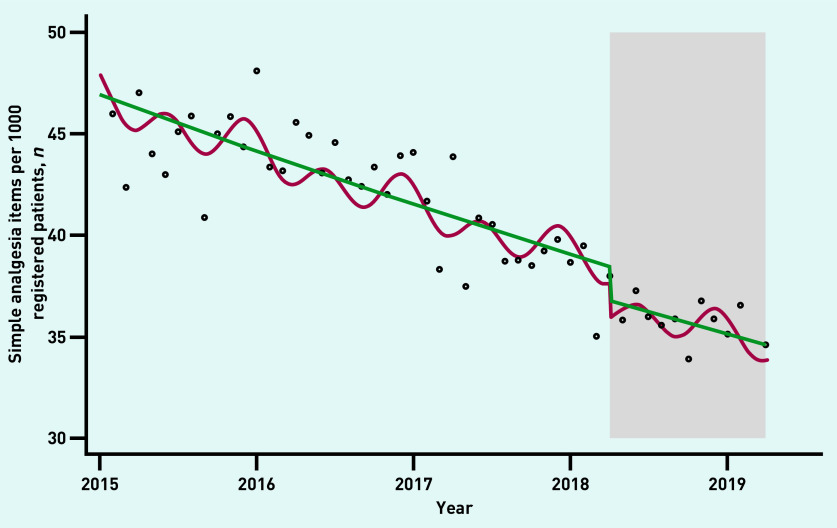
***Seasonally adjusted model of primary care prescribing rates of all simple analgesia (ibuprofen/paracetamol tablets, capsules, and suspensions, and topical NSAIDs) per 1000 registered patients across England from January 2015 to March 2019.****^a^* *^a^****Red line shows the predicted trend based on the seasonally adjusted regression model; green line shows the de-seasonalised trend; and grey box represents the post-intervention period (after March 2018). NSAID = non-steroidal anti-inflammatory drug.***

Data from 7914 practices were included across the study period, covering approximately 120 million prescriptions for oral paracetamol, oral ibuprofen, and topical NSAIDs. When considering all medication groups together, there was a statistically significant reduction in the number of items prescribed per 1000 registered patients per month by GPs in England since the introduction of the NHS England guidance in March 2018 (the intervention; crude prescribing rates 42.3 [before intervention] versus 35.5 [after intervention] per 1000 patients per month). After adjusting for an underlying linear decline in prescribing rates over time and seasonality, the intervention was associated with a statistically significant 4.4% level change reduction in prescribing rates (aIRR 0.96, 95% CI = 0.92 to 0.99, *P* = 0.027, Figure 1). The time- and season-adjusted prescribing rates reduced from 38.5 to 36.6 prescriptions per 1000 per month, from the month before to the month after the intervention.

The ITSAs for each of the subgroups of simple analgesia showed similar trajectories, with all except ibuprofen tablets/capsule, demonstrating a small statistically significant reduction in prescribing rates following the intervention, after accounting for the underlying long-term linear time trend and seasonality (Table 1). The greatest statistically significant level change was seen in ibuprofen suspension (13.2% reduction in prescribing rate, aIRR 0.868, 95% CI = 0.758 to 0.993, *P* = 0.045), and no statistically significant level change was seen in ibuprofen tablets and capsules (aIRR 0.991, 95% CI = 0.931 to 1.055), Table 1. The time series analysis for all individual medication groups can be found in Supplementary Figure S1.

**Table 1.** Effect of intervention on prescribing rates of simple analgesia^a^

**Medication group**	**Reduction, %**	**aIRR**	**95% CI**	***P-*value**	**Pre-intervention slope (by month)**	**Post-intervention slope (by month)**
All simple analgesia	4.4	0.956	0.919 to 0.995	0.027^b^	−0.22	−0.18
Paracetamol tablet/capsule	3.9	0.961	0.925 to 0.999	0.05^b^	−0.15	−0.12
Paracetamol suspension	9.3	0.907	0.827 to 0.995	0.045^b^	−0.02	−0.02
Ibuprofen tablet/capsule	0.9	0.991	0.931 to 1.055	0.772	−0.06	−0.04
Ibuprofen suspension	13.2	0.868	0.758 to 0.993	0.045^b^	−0.01	−0.01
Topical NSAID	9.0	0.910	0.873 to 0.948	<0.001^b^	0.03	0.03

a For all, and for each subgroup of, simple analgesia the percentage reduction in prescribing rates associated with the intervention is given for the time and seasonally adjusted model, along with the aIRR and 95% CIs. The slope coefficients for the linear trends before and after the intervention are shown (as change in prescribing rate per 1000 registered patients per month).

b P *<0.05. aIRR = adjusted incidence rate ratio. CI = confidence interval. NSAID = non-steroidal anti-inflammatory drug.*

**Figure 2.**
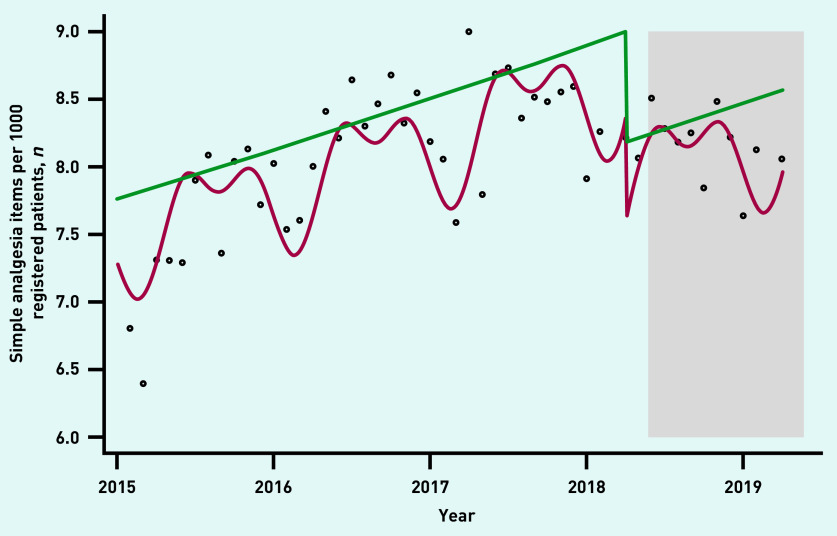
***Seasonally adjusted model of primary care prescribing rates of topical NSAIDs per 1000 registered patients across England, from January 2015 to March 2019.****^a^* *^a^****Red line shows the predicted trend based on the seasonally adjusted regression model; green line shows the de-seasonalised trend; and grey box represents the post-intervention period (after March 2018). NSAID = non-steroidal anti-inflammatory drug.***

The rate of prescriptions for all but topical NSAIDs had begun to decrease before both the date the guidance was published and the related consultation period for all medication groups analysed (Supplementary Figure S1). Indeed, the rate of topical NSAID prescriptions was steadily increasing. Following the intervention, the immediate level change reduction was not sustained, and prescribing has continued to rise again (Figure 2). The average actual spend on simple analgesia per 1000 patients for the 12-month period after the intervention was 98 GBP, compared with 123 GBP in the 12 months before the intervention. It is not possible to separate how much of this is attributable to the intervention rather than to the underlying time trend. However, using the previous 12 months as a baseline, the statistically significant 4.4% reduction in prescribing associated with the intervention equates to an approximate additional reduction of 5.40 GBP per 1000 patients, or approximately a 320 000 GBP saving to the NHS across England for the year.

### Association between prescribing and deprivation

**Figure 3.**
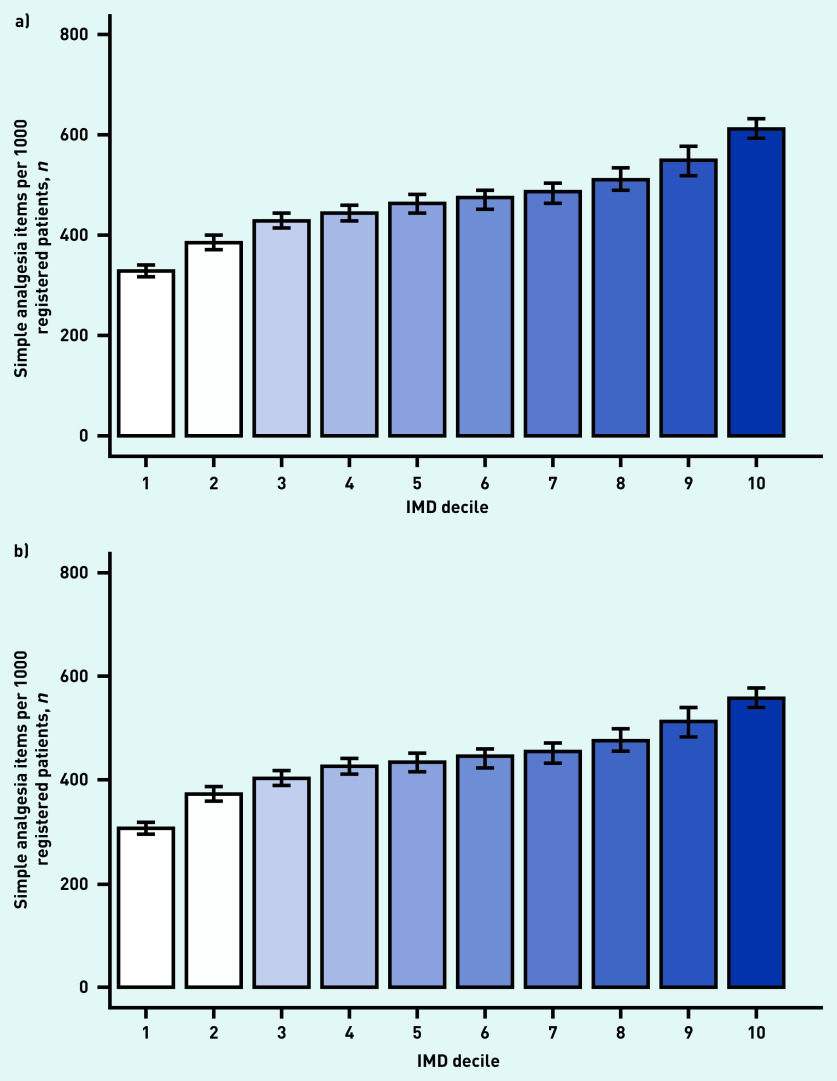
***Average practice prescribing rates of simple analgesia by deprivation decile (a) pre-intervention; and (b) post-intervention.****^a^* *^a^****Deprivation deciles stratified according to practice Index of Multiple Deprivation (IMD)****^11^* ***score. Prescribing rates given as number of items of simple analgesia prescribed per 1000 registered patients over a 12-month period. Error bars show 95% confidence intervals.***

In the 12 months before the intervention, there was a higher rate of prescribing of simple analgesia in more deprived practices (329 items per 1000 registered patients in the least deprived decile versus 612 in the most deprived decile; 709 or 710 practices per decile). In the 12 months after the intervention, this association persisted (Figure 3a and 3b), though there was a general reduction in prescribing rates across all deciles (Supplementary Table S1a).

In a multivariable Poisson regression analysis, in the 12 months before the intervention, the rate of prescribing of simple analgesia was around 2.5 times higher in practices in the most deprived quintile compared with those in the least deprived quintile (aIRR 2.44, 95% CI = 2.33 to 2.57). Similar differences were found in the 12 months after the intervention (aIRR 2.42, 95% CI = 2.30 to 2.56, for the most versus least deprived quintile, Supplementary Table S1b). The geographical variation of prescribing rates by CCG is shown in a choropleth map (Supplementary Figure S2).

### Guidance implementation by clinical commissioning groups

FOI requests were submitted to all 191 CCGs (Supplementary Box S2). Of these, 170 (89%) had a formulary for use by primary care prescribers. A total of 172 (90%) CCGs claimed to have given consideration to the NHS England guidance, with 86 (45%) confirming that they had developed their own policy regarding simple analgesia prescribing (28 had a policy before March 2018). A further 68 (36%) released a ‘position statement’ or directly replicated the NHS England guidance, with 18 (9%) others suggesting that a CCG-specific policy was currently under development (data not shown).

Relevant education for prescribers had been provided by 120 (62%) CCGs. A wide variety of strategies had been used, the most common being electronic or written communications; meetings to discuss the policy; and training sessions (including e-learning). Financial incentivisation is being used by 55 (28%) CCGs, with 26 (14%) indicating plans to enforce the guidance (data not shown).

## DISCUSSION

### Summary

NHS England published guidance for CCGs in March 2018 to encourage primary care prescribers to rationalise the prescription of medications that were also available for purchase over the counter.^5^ Focusing on the impact on simple analgesia prescribing, the authors found that the intervention resulted in a small but statistically significant additional reduction in prescribing rates after accounting for the underlying long-term decline in prescribing and seasonal variation. However, the magnitude of reduction varied with different analgesics, the highest being for ibuprofen suspension. The reasons for this are unclear. Perhaps individuals with short-term self-care conditions that the NHS England guidance targets are more likely to be prescribed suspension ibuprofen, for example, children with acute febrile illness. Formulations that are more likely to be used for longer-term pain management, for example, tablets or capsules may continue to be prescribed in line with the guidance and thus prescribing rates would reduce by a lesser degree than other formulations, such as suspensions, which are less likely to be prescribed for long-term pain management. This could also partly explain the different (increasing) prescribing profiles seen for topical NSAIDs; a prescription for this formulation may be more likely sought for longer-term pain management. There is also the possibility that willingness of patients to purchase simple analgesia over the counter is inversely proportional to the personal cost incurred. Topical NSAIDs are usually more expensive over the counter than tablet or capsule formulations, therefore prescribers may be more willing to provide a script, especially if a patient qualifies for free prescriptions. However, the underlying reasons for this unusual trend require further exploration.

On exploring whether there was any change in the socioeconomic gradient of prescribing before and after publication of the guidance, the authors found no evidence to suggest a widening of the existing inequality of prescribing rates by Index of Multiple Deprivation score decile. Finally, through FOI requests, the authors found CCGs were employing a range of approaches for implementing the guidance, from no implementation to policy development and education.

The authors were unable to examine the effect of implementation measures given the disparity in how and when CCGs implemented this guidance. However, it is unlikely that CCG implementation resulted in the rapid level change in prescribing found in this study. The wide publicity surrounding the guidance publication may have resulted in immediate modification of prescribing behaviours. Indeed, publicity of guidance and publications has been previously noted to be associated with changes in prescribing, though it is difficult to attribute causation.^19^^,^^20^

### Strengths and limitations

The strengths of this study include the inclusion of primary care prescribing across England, with a long lead-in duration before the studied intervention. The analysis of individual CCG implementation measures provides evidence of heterogeneity in actions across the country, and this is an area where further work is required.

There are limitations in the presented study. Aggregated practice-level prescribing data were used so it was not possible to determine the indications for prescriptions. The deprivation analyses were not adjusted for confounders other than age, sex, and practice list size. Individual patient data would be required to identify and account for other factors that may drive prescribing, such as the presence of chronic disease, the incidence of acute febrile illness, and the overall age distribution of the registered patients. Furthermore, the deprivation analyses required the assumption that each practice only had a single deprivation score. Individual-level data analyses are required to confirm whether or not patients from more deprived backgrounds are not disadvantaged by the guidance. A second limitation surrounds the use of ITSA in general: that the level changes in prescribing rates seen may not have been secondary to the publication of the NHS England guidance but rather to other factors. However, most of the level changes seen were statistically significant and the new guidance was widely publicised, so it is possible this influenced prescribing behaviours. Third, this analysis could not ascertain whether the form of CCG implementation influenced prescribing rates.

### Comparison with existing literature

The pre-intervention trend of declining prescribing rates of simple analgesia suggests prior influencing factors. NHS ‘111’ services may have had some impact. In England, the ‘111’ telephone service provides medical advice and signposting to appropriate services. By suggesting treatment plans or pharmacy services, the ‘111’ service may reduce need for patients to seek prescriptions from their GP, and data from the service suggest the frequency of calls taken has increased by about 25% between 2015 and 2019.^21^

No change in the relationship between practice-level socioeconomic deprivation and prescribing rates of simple analgesia before and after the intervention was found, despite prior concerns around health inequalities. This may in part be due to the general exceptions clause in the guidance, with the higher prescribing rate seen in more deprived practices reflecting a higher prevalence of chronic conditions that require simple analgesia.^22^ In practice, the requirement for patients to buy simple analgesia themselves risks the least well off, or vulnerable, in society being unable to purchase or access required medication. The authors cannot exclude the creation of such inequality by this guidance based on the results of the present analysis. Furthermore, health inequalities can occur in domains other than deprivation level, such as ethnicity, and these were not considered in the present analysis of aggregate practice-level data. There is also a risk that shifting purchasing responsibility to patients results in additional inappropriate use of OTC simple analgesics. Indeed, inappropriate use has been described to be a risk of purchasing NSAIDs over the counter, with gaps identified in consumer knowledge,^23^^,^^24^ and it is possible that such outcomes are associated with deprivation.

In addition to the finding that many CCGs were replicating the NHS England guidance as policy, or developing their own, some had used or considered additional strategies for implementation, including education, financial incentives, and enforcement. A systematic review found that educational interventions improved prescribing competency in both medical and non-medical prescribers.^25^ Despite some evidence for their effect,^26^^–^^28^ some have questioned whether the introduction of incentivisation or enforcement may impact the delivery of proper and ethical care.^29^^,^^30^ This notion is particularly concerning here as there are genuine exceptions whereby the prescribing of OTC medications is justified. In addition, it may also leave GPs in breach of their General Medical Services contracts to refuse to prescribe medications outside of the guidance.^31^ As the present analysis did not compare linear prescribing trend changes before and after guidance publication, the authors are unable to make inferences around the effectiveness of different forms of implementation.

### Implications for research and practice

Further work is required to identify which CCG implementation measures bring about the greatest impact on prescribing behaviour. Ultimately, although the promotion of self-care and the use of alternative healthcare avenues may play a key role in medicines optimisation, mere publication of guidance on prescribing restrictions may only result in a modest cost saving to the NHS. CCGs play a key role in ensuring effective implementation, and the value and potential harms of such implementation, including any detrimental effects on the doctor–patient relationship, will need to be the focus of future work.
